# Single oral dose of bovine Fc-fused single-domain antibody enables multi-week serum detectability in neonatal calves

**DOI:** 10.3389/fvets.2026.1827924

**Published:** 2026-04-29

**Authors:** Songyi Yang, Chaerin Kim, Jeongsu Moon, Hanearl Kim, Seonggyu Hong, Iqbal Maulana Taufik, Jian Kim, Soohyun Park, Jeonghee Han, Sungjae Kim

**Affiliations:** 1 College of Veterinary Medicine and Institute of Veterinary Science, Kangwon National University, Chuncheon, Republic of Korea; 2 ADbiotech Co., Ltd., Chuncheon, Republic of Korea; 3 Department of Companion Animal Health, Kyungbok University, Namyangju, Republic of Korea

**Keywords:** Fc fusion, neonatal calf, oral delivery, passive immunization, rotavirus, single-domain antibody

## Abstract

Neonatal calf diarrhea caused by bovine rotavirus (BRV) remains a major health and economic burden in cattle production, and current oral antibody approaches require frequent dosing because of poor systemic persistence. This study investigated whether a single oral dose of a BRV-specific antibody engineered with an immunoglobulin Fc region—designed to extend circulatory half-life—could be detected in the blood of newborn calves for up to 28 days. The antibody construct showed high-affinity binding to BRV antigen and effectively neutralized viral infection in cell culture. In a controlled calf trial, neonatal Holstein calves under 24 h of age (*n* = 5 per group) received a single oral dose of 20 mg of the Fc-fused antibody in milk replacer; control calves received milk replacer alone. Blood samples were collected over 28 days. Treated calves showed numerically higher serum antibody signals than controls throughout the observation period, with statistically significant differences at days 6 and 10. An assay targeting the Fc region provided numerically greater group separation than a general antibody detection format. These results provide proof-of-principle that a single oral dose of an Fc-fused single-domain antibody was systemically detectable for up to 28 days in neonatal calves, supporting the potential for extended dosing intervals compared with conventional oral immunoglobulin strategies.

## 1 Introduction

The immediate postnatal “open-gut” period in ruminants permits nonselective macromolecular uptake across the intestinal epithelium, enabling absorption of colostral immunoglobulins and the establishment of passive immunity in neonatal calves (1, 2). Taking advantage of this physiological window, the present study administered Fc-VHH within 24 h of birth to coincide with peak absorptive capacity. This timing was intended to maximize intestinal uptake of the intact Fc-fused construct before gut closure reduces macromolecular permeability. Because bovine calves are born essentially agammaglobulinemic due to negligible transplacental immunoglobulin transfer, the acquisition of colostral immunoglobulin G (IgG) is critical for early systemic protection against infectious agents (1). This absorptive capacity declines rapidly within the first 24–36 h after birth, and the efficiency of passive immune transfer is influenced by multiple factors, including the timing of first feeding, colostrum quality, and neonatal physiological status (2). Despite improvements in colostrum management, neonatal calf diarrhea caused by bovine rotavirus (BRV) remains an important cause of pre-weaning morbidity and mortality in dairy and beef production systems, and Group A rotavirus has been identified as one of the most prevalent enteric pathogens in diarrheic calves under field conditions (3). In addition, variability in colostral immunoglobulin absorption, driven by periparturient stress, delayed feeding, and inter-individual differences in gut permeability, limits the consistency of colostrum-mediated protection, collectively contributing to the risk of failure of passive transfer under field conditions (4, 5).

Single-domain antibodies (VHHs; also known as nanobodies), comprising the variable domains of camelid heavy-chain-only immunoglobulins, are attractive candidates for oral antiviral prophylaxis because of their small size, biochemical stability, ease of recombinant production, and potent epitope-specific neutralization. BRV-specific VHHs delivered orally have been reported to inhibit infection in calves (6), and orally administered VHHs targeting gastrointestinal pathogens have been recognized as a promising platform for passive immunization (7). The oral route is particularly advantageous in neonatal calves because it is non-invasive and readily compatible with routine colostrum or milk-replacer feeding. However, although VHHs are well suited for enteric targeting, their low molecular mass results in rapid renal clearance, limiting systemic persistence when prolonged circulating exposure is desired (8).

Fusion to an immunoglobulin Fc region is a well-established strategy for improving the pharmacokinetic properties of protein therapeutics. Fc fusion increases hydrodynamic size and enables interaction with the neonatal Fc receptor (FcRn), which rescues IgG from intracellular degradation through pH-dependent recycling and thereby prolongs serum persistence (9–12). FcRn-mediated IgG handling has also been documented in bovine tissue, including the lung, supporting the biological relevance of this pathway in cattle (10). More broadly, Fc-fusion proteins have been widely developed to enhance the stability and half-life of biologic therapeutics (13, 14). FcRn-related studies further support a role for Fc engagement in transepithelial transport and prolonged systemic exposure, including evidence that Fc engineering can improve epithelial transcytosis efficiency (15, 16). In addition, Fc-linked VHH constructs have shown extended *in vivo* protection in preclinical models, supporting the potential of this format to combine the targeting precision of VHH with the pharmacokinetic advantages of IgG Fc (17). Among bovine IgG subclasses, IgG1 was specifically selected as the Fc scaffold for the present construct. In adult bovine serum, IgG1 and IgG2 are present in approximately equal concentrations; however, during the prepartum colostrogenesis phase, IgG1—but not IgG2—is selectively transcytosed across mammary epithelial cells from the maternal circulation into colostrum via a mechanism involving FcRn, resulting in IgG1 concentrations in colostrum that are approximately four- to five-fold higher than in serum (4, 18). Consequently, IgG1 is the predominant immunoglobulin subclass absorbed by the neonatal calf intestine during the open-gut period and the primary isotype mediating early systemic passive immunity (1, 2). The use of bovine IgG1 Fc therefore reflects the most biologically relevant scaffold for a strategy designed to capitalize on FcRn-mediated pharmacokinetic handling in the neonatal bovine host. Based on this rationale, the present study aimed to determine: (i) whether a single oral dose of an anti-BRV VHH fused to bovine IgG1 Fc (Fc-VHH) could achieve systemic detectability for at least 4 weeks in neonatal Holstein calves; and (ii) whether an Fc-specific ELISA readout could provide greater discrimination than a general anti-bovine IgG detection format.

## 2 Materials and methods

### 2.1 Ethics statement and animals

A 10-year-old male alpaca (*Vicugna pacos*) was used for VHH library generation. Ten neonatal Holstein calves (<24 h old) were randomly allocated to a treatment group (oral Fc-VHH, *n* = 5) or a vehicle-control group (milk replacer only, *n* = 5). All calves were admitted within 24 h of birth without prior colostrum intake and were fed milk replacer exclusively throughout the study period, ensuring the absence of endogenous BRV-specific immunoglobulins at baseline. All procedures were approved by the Institutional Animal Care and Use Committee (IACUC) of ARK Resource Co., Ltd. (approval no. AW-21004; approved 2021-01-26).

### 2.2 Immunogen, alpaca immunization, VHH library construction, and phage selection

BRV strain KVCC-VR9200179 (G6P [5]; Korea Veterinary Culture Collection), reflecting the predominant genotype in South Korea, was inactivated by treatment with 0.1% formalin at 37 °C for 72 h; complete inactivation was confirmed by the absence of cytopathic effect on MA104 cells after three blind passages. The alpaca received five subcutaneous injections at 2-week intervals (1 mg antigen per injection; pre-inactivation stock titer: 1.0 × 10^8^ TCID₅₀/mL; dose expressed as total protein mass, as infectious titer is not measurable post-inactivation) using Complete Freund’s Adjuvant (CFA; Sigma-Aldrich, St. Louis, MO, United States) for the primary dose and Incomplete Freund’s Adjuvant (IFA; Sigma-Aldrich) for boosters; seroconversion was confirmed by indirect ELISA; the endpoint titer of anti-BRV IgG reached approximately 1:500,000 after the final immunization, where endpoint titer was defined as the highest serum dilution yielding an OD_450_ value greater than 0.2. Peripheral blood mononuclear cells (PBMCs) were isolated after the final boost; total RNA was extracted with TRIzol™ (Thermo Fisher Scientific, Waltham, MA, United States), reverse-transcribed with SuperScript™ III, and VHH repertoires were amplified by nested PCR. Amplicons were digested with SfiI (New England Biolabs, Ipswich, MA, United States) and cloned into phagemid vector pADL-22c (Antibody Design Labs) to construct a phage-display library comprising 2.5 × 10^7^ independent clones with an insertion rate of 87.5%, as confirmed by colony PCR of randomly selected transformants; sequencing analysis confirmed that the library contained diverse VHH sequences. Biopanning was performed on BRV-coated 96-well plates, and monoclonal high-affinity binders were identified by periplasmic ELISA screening. After the final round of biopanning, individual clones were screened by phage ELISA; clones showing an OD_450_ value ≥3-fold that of the negative control and an absolute OD_450_ value greater than 0.3 were considered positive. To eliminate non-specific binders, phage ELISA was performed against both antigen-coated and negative control wells (BSA-coated and uncoated wells), and clones exhibiting high signals in negative control wells were excluded. Representative phage ELISA screening data for individual clones are provided in Supplementary Figure 1. The top-ranked VHH clone was selected based on the highest signal-to-background ratio and fused in-frame to a bovine IgG1 Fc sequence, transiently expressed in HEK293 cells, and purified from clarified supernatant by Protein A affinity chromatography (HiTrap Protein A HP, Cytiva) under standard binding (PBS, pH 7.4) and elution (0.1 M glycine, pH 3.0, immediately neutralized) conditions, followed by buffer exchange into PBS (pH 7.4); protein concentration was determined by absorbance at 280 nm.

### 2.3 Biochemical characterization

The purified preparation was used for all subsequent biochemical and *in vivo* experiments. SDS-PAGE was performed on 13% Mini-PROTEAN® TGX™ gels (Bio-Rad, Hercules, CA, United States) under non-reducing and reducing (100 mM DTT) conditions (~2 μg protein per lane; 120 V, 90 min) and visualized with Coomassie Brilliant Blue R-250 using Precision Plus Protein™ Dual Color standards (Bio-Rad). For Western blot verification, proteins resolved under the same SDS-PAGE conditions were transferred to a PVDF membrane (Mini Trans-Blot® Cell, Bio-Rad; 100 V, 60 min), blocked with 5% BSA in PBST (0.05% Tween-20) for 1 h at room temperature, and incubated with Peroxidase AffiniPure™ Goat Anti-Bovine IgG, Fc fragment-specific antibody (Jackson ImmunoResearch; 1:20,000) for 1 h 20 min at room temperature. Signals were developed using Pierce ECL Plus Western Blotting Substrate (Thermo Fisher Scientific) and imaged on an ImageQuant LAS 500 system (Cytiva). Molecular weight was estimated using T&I ACCU Prestained Protein Marker (PJM-0605 N; Tech&Innovation, Seoul, Republic of Korea) as a reference standard.

### 2.4 Binding ELISA (EC_50_) and *in vitro* neutralization (IC_50_)

BRV-coated ELISA plates (100 ng/well; inactivated preparation, protein mass as quantitative unit given that post-inactivation infectious titer is not measurable; blocked with 5% skim milk, BD Difco™) were incubated with serial dilutions of Fc-VHH followed by Peroxidase AffiniPure™ Goat Anti-Alpaca IgG VHH domain (Jackson ImmunoResearch, West Grove, PA, United States); EC_50_ values were calculated by four-parameter logistic (4PL) regression (GraphPad Prism v8.0.2; *n* = 3 independent experiments). For the neutralization assay, MA104 cells (ATCC, Manassas, VA, United States) were exposed to pre-formed Fc-VHH-BRV mixtures (two-fold dilution series, 625–0.01 nM) for 1.5 h at 37 °C, washed, and cultured for 72 h. Cell viability was quantified by Cell Counting Kit-8 (CCK-8; Dojindo, Kumamoto, Japan) at 450 nm, and IC_50_ was derived by 4PL regression (*n* = 3).

### 2.5 Oral dosing and serum ELISAs

Within 24 h of birth, treated calves received a single oral dose of 20 mg Fc-VHH (20 mL at 1 mg/mL; the dose and formulation concentration were based on the authors’ prior oral VHH delivery study in neonatal calves (6) and on practical tolerability considerations for neonatal administration) in milk replacer; controls received an equivalent volume of milk replacer alone. Blood was collected on days 0, 3, 6, 10, 15, 20, 23, and 28; serum was separated and stored at −80 °C. Two BRV-coated ELISA formats (100 ng/well; inactivated preparation, protein mass as quantitative unit given that post-inactivation infectious titer is not measurable; 4 °C overnight) were used: (i) general detection with Peroxidase AffiniPure™ Goat Anti-Bovine IgG (H + L) (Jackson ImmunoResearch); and (ii) Fc-specific detection with Peroxidase AffiniPure™ Goat Anti-Bovine IgG, Fc fragment-specific (Jackson ImmunoResearch; 1:20,000). Serum samples were diluted 1:100 in PBST (0.05% Tween-20); TMB reactions were stopped with 2 M H₂SO_4_ and read at 450 nm.

### 2.6 Statistical analysis

Longitudinal outcomes were analyzed by two-way repeated-measures ANOVA (between-subject factor: group; within-subject factor: day), with the Greenhouse–Geisser correction applied where sphericity was violated and log-transformation applied as needed. *Post hoc* comparisons used Tukey’s test (*α* = 0.05). Receiver operating characteristic (ROC) analyses were performed at the calf level (to avoid pseudoreplication) to evaluate assay-level discrimination between treated and control groups. All analyses were performed in GraphPad Prism v8.0.2.

## 3 Results

### 3.1 Biochemical characterization of Fc-VHH

**Figure 1:**
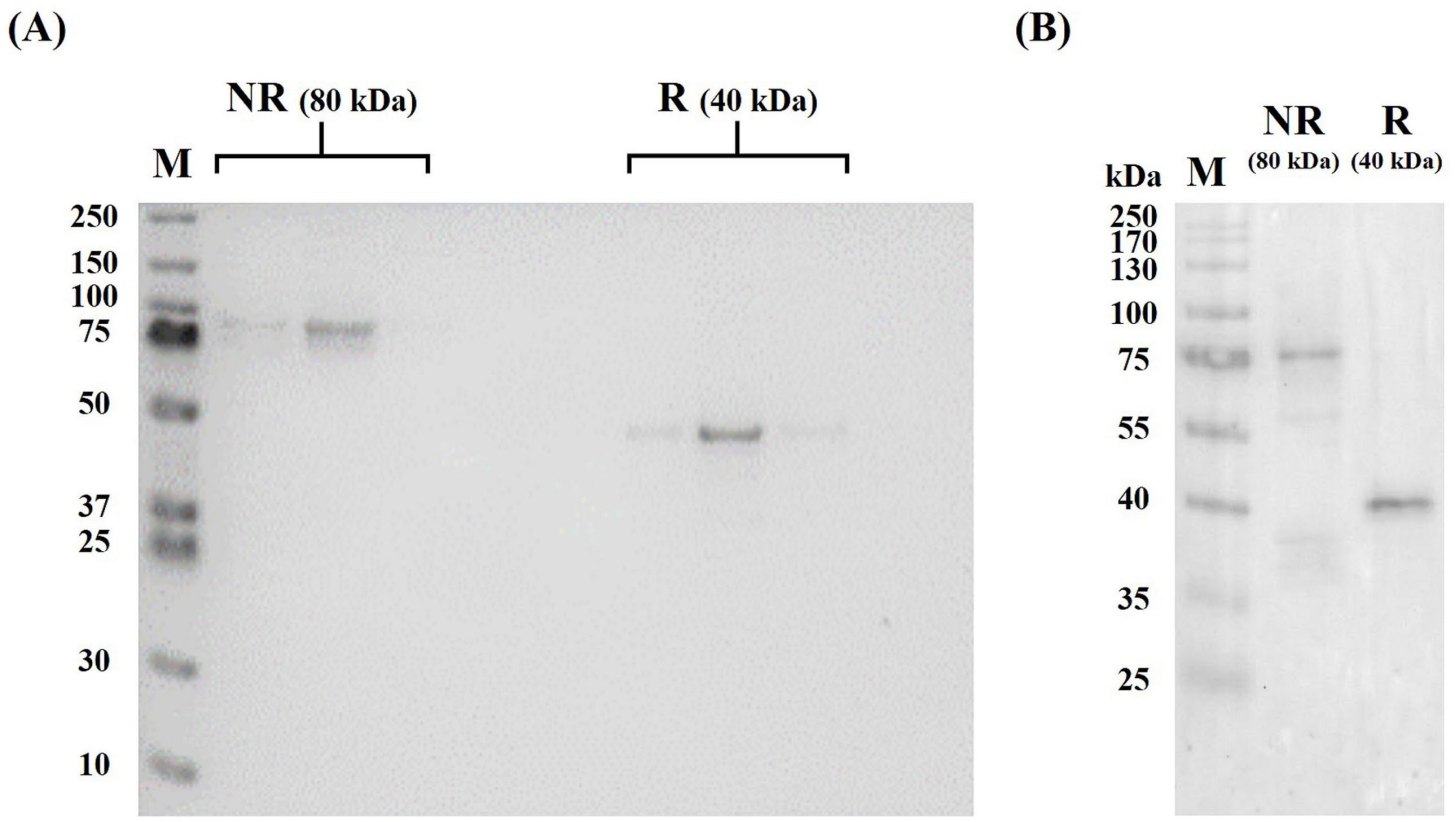
Biochemical characterization of purified Fc-VHH. **(A)** SDS-PAGE under non-reducing (NR) and reducing (R) conditions. Fc-VHH migrated as an approximately 80 kDa dimer under non-reducing conditions and as an approximately 40 kDa monomer under reducing conditions, consistent with Fc-mediated disulfide-linked homodimerization. **(B)** Western blot using anti-bovine IgG Fc fragment-specific antibody confirming the presence of the bovine IgG1 Fc moiety. Lane M: **(A)** Precision Plus Protein™ Dual Color standards (Bio-Rad); **(B)** T&I ACCU Prestained Protein Marker (Tech&Innovation); NR, non-reducing; R, reducing.

SDS-PAGE demonstrated a single predominant band at approximately 80 kDa under non-reducing conditions and approximately 40 kDa under reducing conditions (Figure 1A), consistent with Fc-mediated disulfide-linked homodimerization. No substantial degradation products or high-molecular-mass aggregates were detected, confirming preparation homogeneity. Western blot analysis using an anti-bovine IgG Fc fragment-specific antibody confirmed the identity of the construct, with bands detected at approximately 80 kDa under non-reducing conditions and approximately 40 kDa under reducing conditions (Figure 1B), consistent with the homodimeric and monomeric forms of the Fc-VHH construct, respectively.

### 3.2 Antigen binding and *in vitro* neutralization

**Figure 2:**
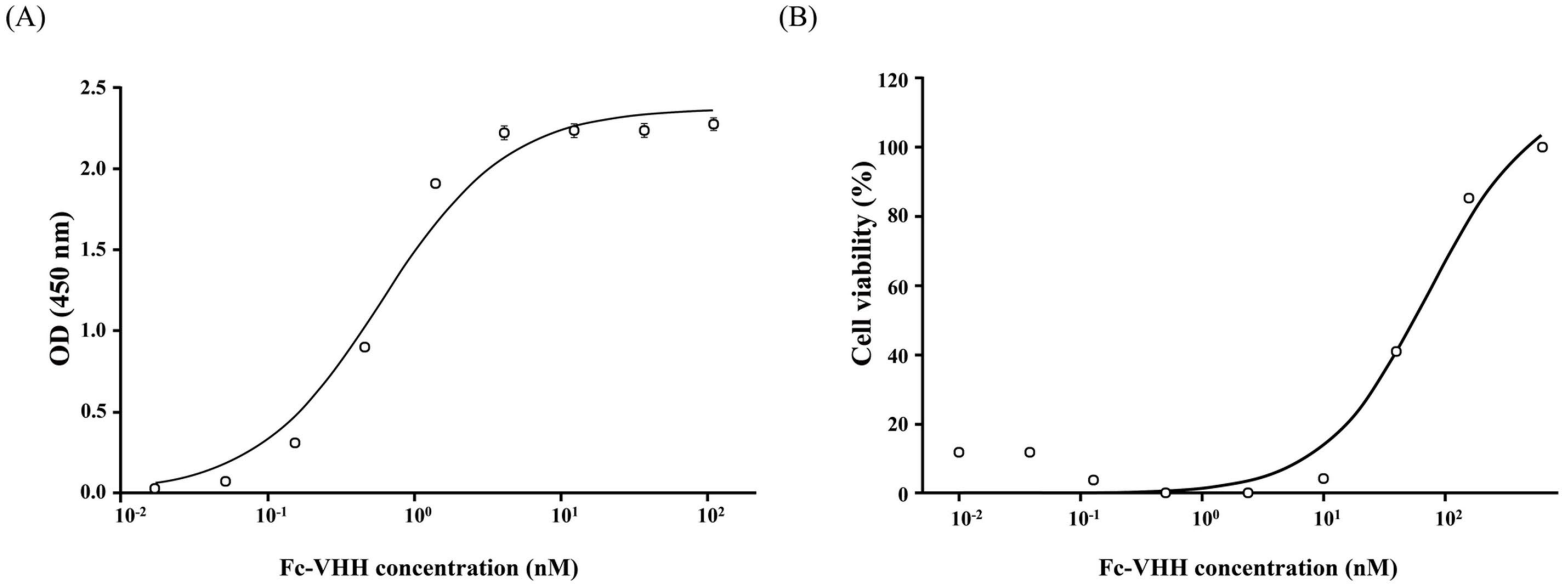
*In vitro* functional characterization of Fc-VHH. **(A)** BRV antigen-binding curve by ELISA. EC_50_ = 0.57 ± 0.02 nM (mean ± SE; 95% CI, 0.49–0.66 nM; *n* = 3 independent experiments) by 4PL regression. **(B)** BRV neutralization in MA104 cells assessed by CCK-8 viability assay. IC_50_ = 52.66 ± 1.94 nM (95% CI, 47.33–57.99 nM; *n* = 3; error bars = SE). Crystal-violet staining confirmed preserved monolayer integrity at concentrations ≥39 nM.

Dose-dependent ELISA binding to BRV antigen yielded an EC_50_ of 0.57 ± 0.02 nM (95% CI, 0.49–0.66 nM) (Figure 2A), within the sub-nanomolar range typically reported for high-affinity VHH scaffolds (8). In the cytopathic effect (CPE)-inhibition assay, crystal-violet staining showed preserved MA104 monolayer integrity at Fc-VHH concentrations ≥ 39 nM, and CCK-8 readouts confirmed concentration-dependent viability restoration (Figure 2B). The IC_50_ was 52.66 ± 1.94 nM (95% CI, 47.33–57.99 nM; *n* = 3).

### 3.3 Serum detectability after oral administration

**Figure 3:**
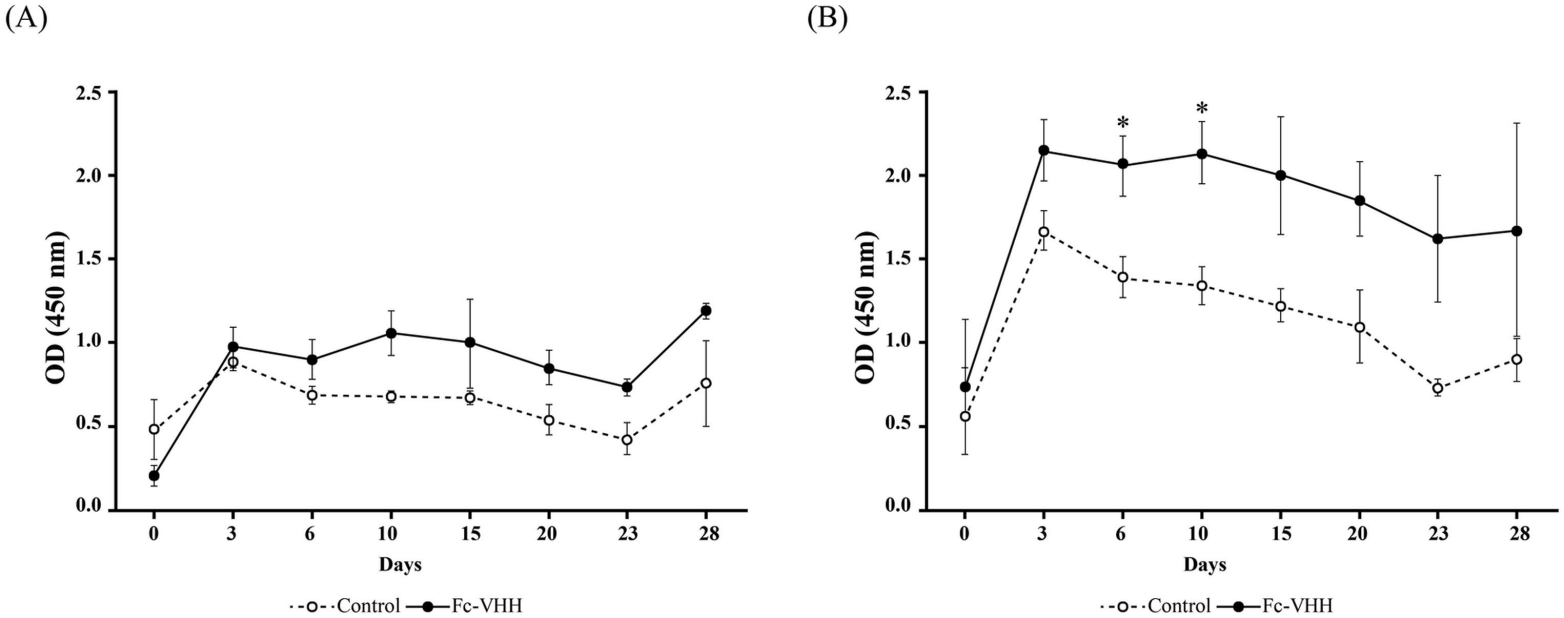
Serum antibody signals following a single oral dose of Fc-VHH (20 mg) in neonatal calves (*n* = 5 per group; days 0–28). **(A)** General anti-bovine IgG (H + L) ELISA. **(B)** Fc-specific ELISA. Treated calves showed elevated OD_450_ values relative to vehicle-controls through day 28. Statistically significant between-group differences were detected on days 6 and 10 (*p < 0.05 by two-way repeated-measures ANOVA with *post hoc* Tukey test). Differences on days 3 and 20 did not reach conventional significance (0.05 ≤ *p* < 0.10). Error bars indicate SE.

Following a single oral dose of 20 mg Fc-VHH, serum ELISA signals in treated calves were consistently elevated relative to vehicle controls across the full 28-day observation period (Figures 3A,B). Two-way repeated-measures ANOVA with *post hoc* Tukey’s testing revealed statistically significant between-group differences at day 6 (*p* = 0.027) and day 10 (*p* = 0.009), with a trend toward significance at day 3 and day 20 (0.05 ≤ *p* < 0.10). No significant differences were detected at other time points. The Fc-specific ELISA followed a similar temporal profile to the general IgG readout but showed numerically greater between-group separation (Figure 3B vs. Figure 3A).

### 3.4 Assay-level discrimination: ROC analysis

**Figure 4:**
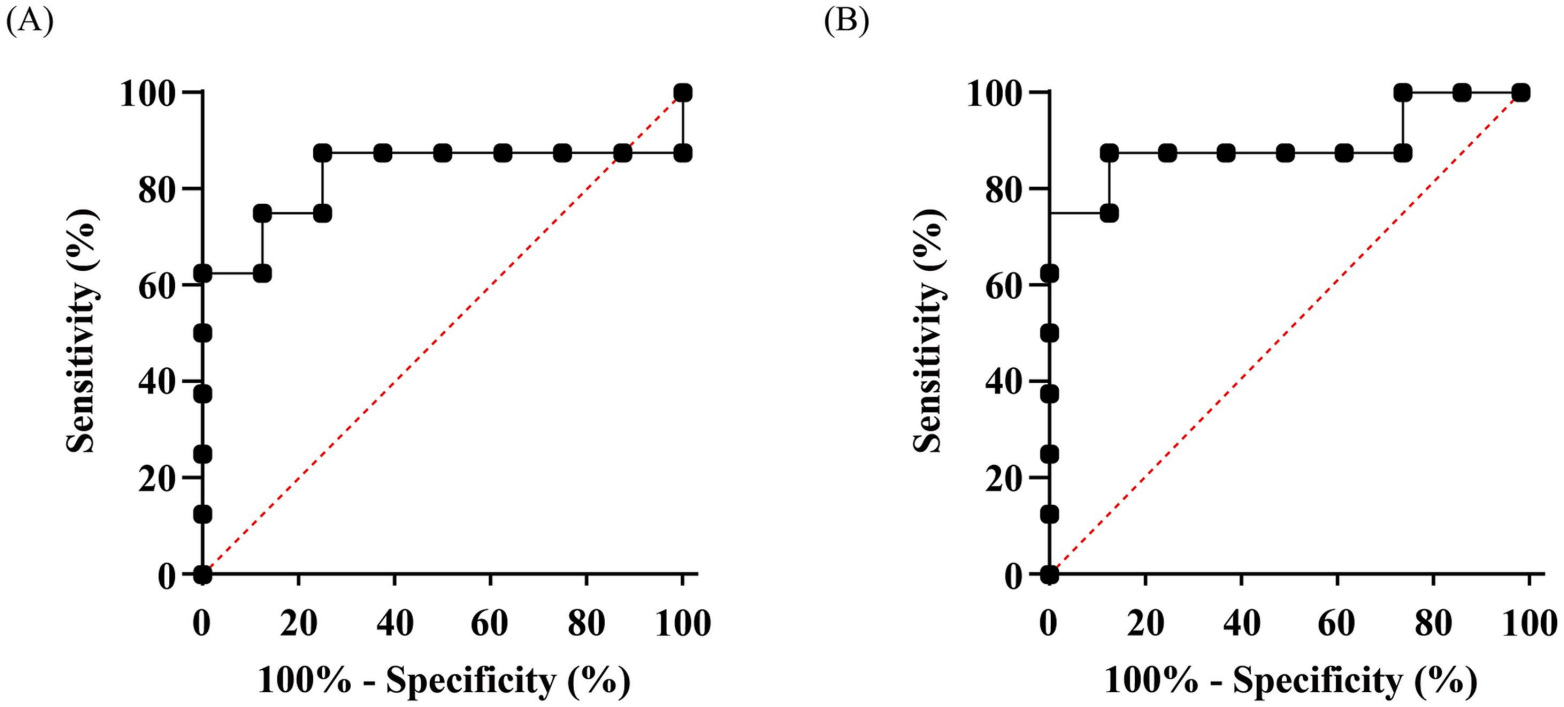
ROC curves evaluating serum ELISA formats for discrimination between Fc-VHH-treated and vehicle-control calves. **(A)** General IgG (H + L) ELISA: AUC = 0.8281 (95% CI, 0.5916–1.000; *p* = 0.0274). **(B)** Fc-specific ELISA: AUC = 0.8906 (95% CI, 0.7075–1.000; p = 0.0087). Both AUCs were significantly greater than 0.5. No formal between-curve statistical comparison was pre-specified. Analyses were performed at the calf level (*n* = 5 per group; 8 serial time points per calf) to avoid pseudoreplication.

ROC analyses performed at the calf level demonstrated measurable discrimination for both ELISA formats (Figure 4). The Fc-specific ELISA achieved an area under the curve (AUC) of 0.8906 (95% CI, 0.7075–1.000; *p* = 0.0087), while the general anti-bovine IgG (H + L) ELISA yielded an AUC of 0.8281 (95% CI, 0.5916–1.000; *p* = 0.0274). Both AUCs were significantly greater than 0.5. No formal between-curve comparison was pre-specified, but the Fc-specific format showed numerically superior group separation.

## 4 Discussion

This study provides proof-of-principle evidence that a single oral dose of Fc-VHH was associated with multi-week systemic detectability in neonatal Holstein calves. Compared with vehicle controls, significant between-group differences in serum ELISA signals were observed on days 6 and 10, and numerically higher signals were maintained through day 28. In the context of neonatal calf immunoprophylaxis, these findings are notable because the early postnatal period is characterized by both high susceptibility to enteric infection and a transient capacity for macromolecular absorption across the intestine (1, 2). Given that BRV remains an important cause of neonatal calf diarrhea under field conditions (3), the present data support further investigation of Fc-VHH as a potential oral platform for extending the duration of passive antibody exposure during this vulnerable period.

The biochemical characterization of the construct supports the plausibility of its *in vivo* behavior. Under non-reducing conditions, the Fc-VHH migrated at the expected size for an Fc-mediated homodimer, whereas reducing conditions yielded the corresponding monomeric band, consistent with disulfide-linked dimerization through the Fc region. The identity of the bovine IgG1 Fc moiety was further confirmed by Western blot analysis, providing direct molecular evidence that the purified preparation contains the intended Fc domain rather than a co-migrating contaminant of similar molecular weight. This structural format is relevant because, although the compact size of monomeric VHHs is advantageous for mucosal targeting, it also contributes to rapid renal clearance and limited systemic persistence (8). Fusion to an Fc domain increases hydrodynamic size and is a widely used strategy for improving the pharmacokinetic performance of protein therapeutics (13, 14). Importantly, Fc fusion did not appear to compromise antigen recognition or antiviral function in the present construct, as indicated by the sub-nanomolar ELISA binding response and preserved neutralizing activity *in vitro*. This interpretation is consistent with previous reports showing that VHH-based molecules can retain biological activity after molecular reformatting and that Fc-linked VHH constructs can provide improved *in vivo* durability (17).

A biologically plausible explanation for the prolonged systemic detectability observed here is that the Fc-VHH construct benefited from both neonatal intestinal uptake and Fc-dependent post-absorptive handling. In calves, the immediate postnatal “open-gut” period permits absorption of intact colostral immunoglobulins and is central to the establishment of passive immunity during early life (1, 2). Although the present Fc-VHH was administered experimentally rather than maternally derived, the same developmental window may have favored uptake of the intact Fc-fused molecule after oral administration. Once absorbed, interaction of the Fc moiety with FcRn may have contributed to systemic persistence by rescuing the construct from intracellular degradation and promoting recycling, as has been well established for native IgG and Fc-containing biologics (9, 11, 13, 14). FcRn-related studies further indicate that Fc engagement can influence transepithelial transport and systemic exposure, and engineered Fc-containing molecules can show enhanced epithelial transcytosis in experimental systems (15, 16). In addition, FcRn expression has been documented in bovine tissue, including the lung, supporting the biological relevance of Fc-mediated IgG handling pathways in this species (10). However, these interpretations remain inferential in the context of the present study. Because FcRn dependence, intestinal transport kinetics, and tissue distribution were not directly assessed, the current findings should be interpreted as consistent with, but not conclusive for, FcRn-mediated uptake and recycling.

The assay comparison also provides useful methodological insight. The Fc-specific ELISA yielded a numerically higher AUC than the general anti-bovine IgG (H + L) format, suggesting that Fc-directed detection may provide improved analytical discrimination for exogenously administered Fc-VHH. A plausible explanation is that the Fc-specific format reduced interference from endogenous bovine immunoglobulins present in neonatal serum, thereby improving the signal-to-background ratio. In the present study, the potential confound of endogenous BRV-specific IgG was further mitigated by the experimental conditions: all calves were confirmed colostrum-free at enrollment and fed milk replacer exclusively throughout the study period. It is well established that colostrum-deprived neonatal calves lack detectable pathogen-specific circulating antibodies at baseline (19), as bovine calves are essentially agammaglobulinemic at birth due to the absence of transplacental immunoglobulin transfer (1). Under these conditions, the BRV-antigen-coated ELISA format selectively captures only the exogenously administered Fc-VHH, as no endogenous BRV-specific bovine IgG was present to contribute background signal. This interpretation is biologically reasonable given the abundance of native bovine IgG in calves after colostrum intake (1, 4), although the present study was not designed to formally validate analytical specificity or to compare assay formats under a pre-specified statistical framework. Accordingly, the ROC findings should be regarded as supportive rather than definitive, and future pharmacokinetic studies would benefit from validated Fc-specific immunoassays and orthogonal quantitative methods capable of measuring absolute circulating Fc-VHH concentrations.

From a translational perspective, the present findings address an important limitation of existing oral antibody approaches for neonatal calf diarrhea. Passive oral immunization is attractive because it is non-invasive and compatible with routine feeding practices in newborn calves, and orally delivered VHHs have already shown promise against BRV and other gastrointestinal pathogens (6, 7). However, conventional oral antibody preparations are generally intended to act within the intestinal lumen and often require repeated administration to maintain coverage (7). In contrast, the present Fc-VHH format may offer a dual advantage: preservation of the practical benefits of oral delivery together with the potential for prolonged systemic persistence conferred by Fc fusion (13, 14). In a field setting, such durability could reduce dosing frequency and improve feasibility, particularly during the first days of life when passive protection is most critical yet naturally variable because of differences in colostrum quality, feeding timing, and neonatal stress (4, 5). Although protection against clinical disease was not tested here, the ability to detect the construct systemically for multiple weeks after a single dose supports the rationale for further development of Fc-VHH-based oral immunoprophylaxis in calves.

The present study has several limitations that should be considered when interpreting the findings. First, only a single dose level was evaluated in a small cohort, precluding robust characterization of dose–exposure relationships, inter-animal variability, or minimum exposure thresholds associated with biological activity. Second, serum ELISA signals provide indirect evidence of systemic detectability but do not permit estimation of absolute pharmacokinetic parameters such as Cmax, terminal half-life, or oral bioavailability. Third, not all post-dose time points showed statistically significant between-group differences, indicating that the duration and magnitude of systemic exposure should not be overinterpreted from the present dataset. The absence of significance at later time points (days 15, 20, 23, and 28) may reflect progressive degradation of the absorbed construct, increasing immune complex formation as *de novo* endogenous antibody responses develop over the course of the study period, whether through natural maturation of the humoral immune system or through active responses to the administered construct, or a dilution effect associated with increasing blood volume as calves grow; the small group size (*n* = 5 per group) also limits statistical power at individual time points. Nonetheless, the numerically elevated signals consistently observed in treated animals through day 28 suggest that biological detectability persisted beyond the window of statistical significance. Fourth, no viral challenge model was included, so it remains unknown whether multi-week systemic detectability translates into clinically meaningful protection against BRV infection or diarrhea. This point is particularly important because prior work has shown that orally delivered BRV-specific VHH can inhibit infection in calves (6), but the relationship between systemic detectability and protective efficacy has not yet been defined. The *in vitro* neutralization assay did not include an irrelevant VHH-Fc fusion or Fc-only protein as a negative control; consequently, non-specific effects of the Fc domain on cell viability or viral infectivity cannot be formally excluded. The concentration-dependent viability restoration observed across a wide dilution range and the preserved MA104 monolayer integrity at concentrations ≥39 nM are consistent with antigen-specific neutralization, but inclusion of appropriate negative controls in future studies will be necessary to confirm that the observed activity is attributable specifically to the VHH domain. Finally, the current study did not examine anti-drug antibody responses, gastrointestinal degradation kinetics, or cross-genotype activity against BRV strains beyond the genotype tested, all of which will be important for future translational development.

Future studies should therefore move beyond proof-of-concept detectability and address mechanism, pharmacokinetics, and efficacy in an integrated manner. In particular, studies incorporating dose escalation, dense sampling schedules, validated quantitative assays, and controlled BRV challenge models will be needed to define exposure–response relationships and determine whether Fc-mediated systemic persistence improves prophylactic performance. Mechanistic experiments evaluating FcRn involvement, intestinal transport, and tissue distribution would also help clarify whether the neonatal absorptive window and Fc-dependent recycling jointly account for the observed systemic detectability (1, 2, 9, 11, 15, 16). In addition, formulation strategies that improve gastrointestinal stability may further enhance the practical utility of this platform for neonatal administration (7, 16).

In conclusion, a single oral dose of Fc-VHH resulted in multi-week systemic detectability in neonatal calves, and the Fc-specific ELISA showed numerically greater group discrimination than a general anti-bovine IgG detection format. Although the underlying transport mechanism and the relationship to protective efficacy remain to be established, these findings support further evaluation of Fc-VHH as a candidate platform for extended-interval oral immunoprophylaxis against neonatal calf diarrhea and justify additional mechanistic, pharmacokinetic, and efficacy studies.

## Data Availability

The original contributions presented in the study are included in the article/Supplementary material, further inquiries can be directed to the corresponding authors.

## Supplementary material

SUPPLEMENTARY FIGURE 1 Monoclonal phage ELISA screening of individual VHH clones against BRV antigen. Individual clones isolated after the final round of biopanning were screened by phage ELISA on BRV-coated plates. OD_450_ values are shown for each colony. The two panels display consecutive clone numbers from the same screening round. The dashed line indicates the positive cutoff threshold (OD_450_ ≥3-fold that of the negative control). Clones exceeding the cutoff were considered candidate binders and subjected to further validation. The arrow indicates the top-ranked clone selected for Fc fusion.
