# Supplementary figures and images for: Single oral dose of bovine Fc-fused single-domain antibody enables multi-week serum detectability in neonatal calves
Source: Front Vet Sci. 2026 Apr 29;13:1827924. doi: 10.3389/fvets.2026.1827924 (PMC13168033; doi:10.3389/fvets.2026.1827924)

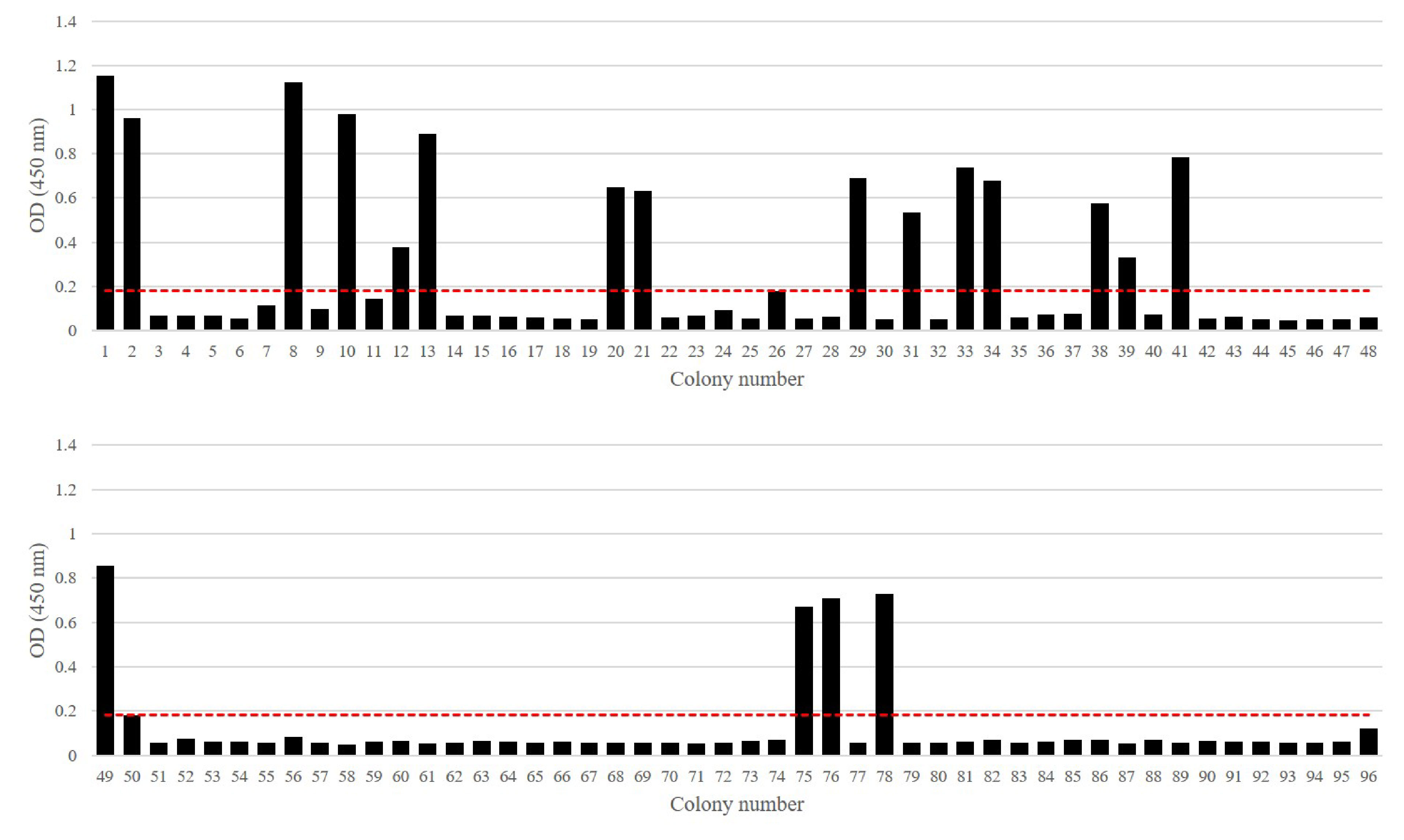

Supplement: SUPPLEMENTARY FIGURE 1 — Monoclonal phage ELISA screening of individual VHH clones against BRV antigen. Individual clones isolated after the final round of biopanning were screened by phage ELISA on BRV-coated plates. OD450 values are shown for each colony. The two panels display consecutive clone numbers from the same screening round. The dashed line indicates the positive cutoff threshold (OD450 ≥3-fold that of the negative control). Clones exceeding the cutoff were considered candidate binders and subjected to further validation. The arrow indicates the top-ranked clone selected for Fc fusion. [file Image_1.jpeg]
